# Core Professionalism Education in Surgery: A Systematic Review

**DOI:** 10.4274/balkanmedj.2017.0534

**Published:** 2018-03-15

**Authors:** Akile Sarıoğlu Büke, Özlem Sürel Karabilgin Öztürkçü, Yusuf Yılmaz, İskender Sayek

**Affiliations:** 1 Emeritus Professor of Paediatric Surgery, Pamukkale University School of Medicine, Denizli, Turkey; 2 Department of Medical Education, Ege University School of Medicine, İzmir, Turkey; 3 Emeritus Professor of Surgery, Hacettepe University School of Medicine, Ankara, Turkey

**Keywords:** Education, residency, medical, surgical professionalism, systematic review

## Abstract

**Background:**

Professionalism education is one of the major elements of surgical residency education.

**Aims:**

To evaluate the studies on core professionalism education programs in surgical professionalism education.

**Study Design:**

Systematic review.

**Methods:**

This systematic literature review was performed to analyze core professionalism programs for surgical residency education published in English with at least three of the following features: program developmental model/instructional design method, aims and competencies, methods of teaching, methods of assessment, and program evaluation model or method. A total of 27083 articles were retrieved using EBSCOHOST, PubMed, Science Direct, Web of Science, and manual search.

**Results:**

Eight articles met the selection criteria. The instructional design method was presented in only one article, which described the Analysis, Design, Development, Implementation, and Evaluation model. Six articles were based on the Accreditation Council for Graduate Medical Education criterion, although there was significant variability in content. The most common teaching method was role modeling with scenario- and case-based learning. A wide range of assessment methods for evaluating professionalism education were reported. The Kirkpatrick model was reported in one article as a method for program evaluation.

**Conclusion:**

It is suggested that for a core surgical professionalism education program, developmental/instructional design model, aims and competencies, content, teaching methods, assessment methods, and program evaluation methods/models should be well defined, and the content should be comparable.

Professionalism is briefly defined as a “social contract” with the society, and is a crucial element of medical practice along with the healing role (1). One of the driving forces for defining professionalism in medicine and surgery was a response to the rapid expansion of the concept of commercialism and associated ethics in these professions. Defining professionalism for medical specialties remains somewhat controversial, but it has been proposed that the main professional values include altruism, respect, honesty, integrity, dutifulness, honor, excellence, and accountability (1,2). The definition of professionalism in medicine has been developed by the American Board of Internal Medicine Foundation, American College of Physicians Foundation, and European Federation of Internal Medicine and published as “Medical Professionalism in the New Millennium: A Physician Charter”. Various countries, professional associations, and councils have studied and accepted this code of professionalism (3,4,5,6,7,8). In addition, university project groups have been established, and conferences have been held on topics, such as the concept of professionalism and its implications in surgical practice and implementation of professionalism education in the field of surgery (9,10,11,12). However, despite all the studies on professionalism, defining it remains challenging. Although professionalism education is one of the major concerns in medical and surgical training programs worldwide, the implementation and assessment of these programs remain debatable (13,14,15,16). It has been suggested that professionalism education includes character development, and implementation of a professionalism education program has been described as designing to educe latent wisdom” (17,18). Parker et al. (19) proposed an orientation program and commitment to professionalism in their Pyramid of Professionalism education model which was tailored for medical students. To initiate an individual’s commitment to professionalism, we defined for the Core Professionalism Education Program (COPEP), which is a start-up program for the development of an individual’s professional values. A comprehensive surgical education program should start with COPEP and integrate professionalism throughout the educational period. COPEP can be planned for surgical residents as well as medical students. The aim of this systematic review was to evaluate studies on COPEP in surgery (COPEPS) for surgical residents.

## MATERIALS AND METHODS

We searched for articles on COPEPS that described a professionalism program conducted during surgical training, including program development model/instructional design methods, aims and competencies, methods of teaching, methods of assessment, and program evaluation model/method. COPEPSs that targeted surgical residents and possessed at least three of the abovementioned characteristics were included.

### Review group

The study review group comprised four members: an emeritus member of the academic staff in Pediatric Surgery who was also a philosophiae doctor student in Medical Education, member of the academic staff in Medical Education, instructional designer in Medical Education, and emeritus member of the academic staff in Surgery who was the chair of the Association for the Evaluation and Accreditation of Medical Education Programs in Turkey.

### Search strategy

A detailed search was conducted between February 2015 and April 2015, and articles published between January 2005 and January 2015 were included. The start date was chosen as 1 year after the Surgical Task Force on Professionalism began in 2003. The Medical Subject Headings (MeSH) words and search strings used are presented in Table 1. Electronic databases searched included EBSCOHOST, PubMed, Science Direct, and Web of Science. The word “professionalism” was not present in the PubMed MeSH vocabulary; hence, a suggestion was made to National Institutes of Health for its inclusion. The research material was collected in Mendeley under separate folders according to the databases searched and time of search. We also searched the web for gray literature, such as proceedings and congress papers. In addition, a separate manual search of the Journal of Surgical Education, Medical Teacher, Medical Education, Academic Medicine, and Clinical Teacher was conducted. References from 11 outstanding articles and reviews were also manually searched (20).

### Selection of eligible articles

The articles were reviewed to select the eligible ones. Articles including at least three components of COPEPS (program developmental model/instructional design method, aims and competencies, methods of teaching, methods of assessment, or program evaluation model/method) were included. Furthermore, both qualitative and quantitative reports were included. Articles that described COPEPSs associated with core surgical professionalism education programs for residents and for others were also included. Articles published in languages other than English and those related to only nonsurgical residency, undergraduate medical education, and continuing medical education programs were excluded. Articles based on programs that claimed of teaching professionalism as a component of other competencies and/or teaching other competencies, such as technical skills (i.e., integrated programs) (21,22,23,24,25,26,27,28,29,30,31,32,33,34,35,36,37,38); those based on programs that taught only one component of professionalism, such as leadership (39,40,41); and those based on programs with only one component of program development or instructional design, such as assessment (22,23,42,43,44,45,46,47,48) were also excluded. Data were extracted and collated into an Excel program by the first reviewer. The articles were then rechecked by the first reviewer. Next, the selected articles were separately reviewed by two reviewers to screen eligible articles. Intra- and inter-rater agreements were calculated.

## RESULTS

**Figure 1:**
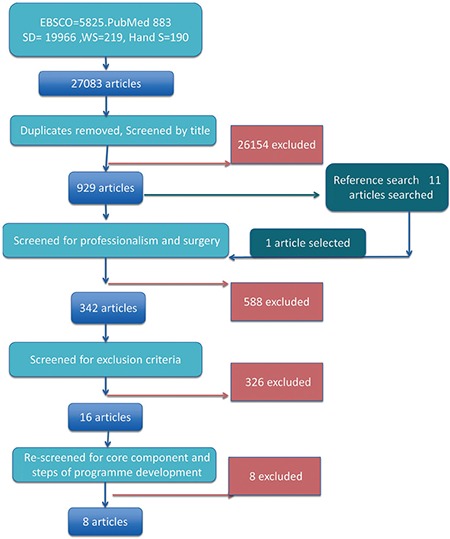
Flowchart of the systematic review.

A total of 27.083 articles were retrieved from EBSCOHOST, PubMed, Science Direct, Web of Science, and manual search (Table 2). A flow diagram of the systematic review is presented in Figure 1. After eliminating duplicates and screening the articles by titles, the remaining 930 articles were screened for those based on professionalism and surgery. As a result, 342 articles were selected. Then, these articles were reviewed for the exclusion criteria. The first reviewer analyzed 342 articles twice in the given time range and identified 16 artciles that reported on COPEPS. Intra-rater agreement for the first reviewer was 97.07% (Cohen’s kappa = 0.532). The 16 articles selected were reviewed again by the two reviewers to select the articles containing at least three components of COPEPS. To ensure reliability throughout the research, intra- and inter-rater reliability measurements were evaluated. The inter-rater reliability results showed a high level of agreement between the two reviewers (Cohen’s kappa = 0.625), indicating that there was an acceptable agreement between the two reviewers for most articles (49,50). Eight articles (9,10,51,52,53,54,55,56) met the research criteria and were analyzed to delineate COPEPS. Three of these reported a component shared with COPEPs for other residents, faculty, nurses, and students. Details regarding the analysis of the eight articles are presented in Table 3.

## DISCUSSION

We concluded that COPEPS is necessary to orient surgical residents toward the principles of surgical professionalism. The residents are expected to understand that professionalism is a social contract and in fact their social contract starts as soon as they start working as a professional. The implementation of this core program is therefore crucial. In this systematic review, we identified eight articles on COPEPS that met the review criteria. These selected articles covered programs specific to COPEPS during surgical residency. A combined core component targeting faculty, residents, nurses, and medical students was detected in addition to the core component for surgical residents (54,55,56). These combined programs could be useful to nurture teamwork, but the authors suggested that the core component for surgical residents should not be neglected. Only one article covered the instructional design method, describing the Analysis, Design, Development, Implementation, and Evaluation model (54). All the selected articles prescribed a method for COPEPS, although a particular method was not specified. Six articles specified the Accreditation Council for Graduate Medical Education (ACGME), whereas one specified the nine domains of Royal Australian College of Surgeons as COPEPS criteria. Because it is difficult to define professionalism, we suggest that the baseline criteria should be used as a guide for developing COPEPS. Even when the aims and competencies of COPEPS were based on those specified by ACGME, a wide range of differences were observed in their content, except two consecutive articles describing the same program. We suggest that it is better to develop COPEPS on the basis of criteria that are universal or associated with a national professional association or content that completely defines professionalism, and should be comparable. The duration of the implemented programs described in the articles varied from hours to a year. Hochberg et al. (53) preferred to provide core professionalism education throughout the year, including surgical residents in their first, second, and third years of training simultaneously (9,53). Another important point is to provide COPEPS at the beginning of surgical residency. In the following years, COPEPS could be supported by integrated surgical professionalism education or refresher programs. We believe that for COPEPS, both conditions can be applied, depending on the requirements of surgical residents, provided that the basic principles of professionalism education are followed. The most common teaching methods described were role modeling and scenario- and case-based learning. Other methods included conferences using multisource feedback and learning analysis, discussion, reflection, self-reflection, debriefing, mentorship, self-awareness exercises, team-building activities, role play, journal and book clubs, grand round presentations, and objective-structured clinical examination (OSCE). Other teaching methods included the use of simulated environments, simulated patients, mannequins, videotapes, and video clips. A recently published article also described the use of flipped classrooms. It has been reported that role modeling and mentoring were the most frequently used teaching methods for professionalism education (57,58). The evaluation of professionalism is another important issue (16,18,43,59). The methods of evaluation reported in the articles included OSCE, 360° evaluation, self-assessment, national survey, patient complaint and satisfaction survey, standard patient questionnaires, and multiple-choice questions. However, if surgical residents are expected to develop professional behavior and attitude, it would not be appropriate to use only multiple-choice tests and questionnaires for evaluation. The evaluation of a professionalism education program is also extremely important. The Kirkpatrick model of program evaluation was used in one of the selected articles (60). Other reported methods included questionnaires, 360° evaluation, and pre- and post-test designs.

The lack of consensus regarding professionalism education can be regarded as a limitation of this study (13).

Teaching professionalism needs a cognitive base, but environment is also important; hence, principles from both cognitive and situated learning theories should be applied (57). Some institutions have tried to achieve this by establishing an institutional culture or ecology of professionalism within the institution through professionalism improvement activities (61,62,63). Surgical residents interact with other people as a part of a complex system, and education is imparted everywhere. Thus, a core or even an integrated professionalism education program would not be sufficient; it would be better to cultivate a culture of professionalism throughout the clinic and institution. We detected that a complete professionalism education program for surgical residents begin with COPEPS, and that COPEPS should end with a written commitment. Additionally, COPEPS should include the steps of instructional design and should be well defined and elaborately constructed in accordance with the regulations of national and international associations.

## Figures and Tables

**Table 1:**
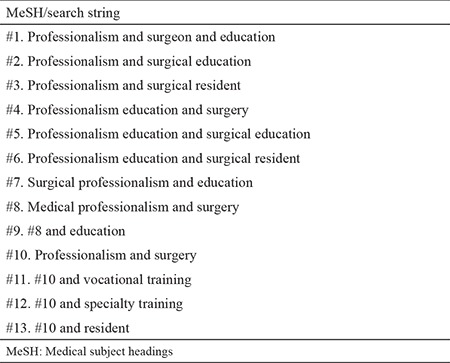
MeSH words and search strings used in the literature search

**Table 2:**
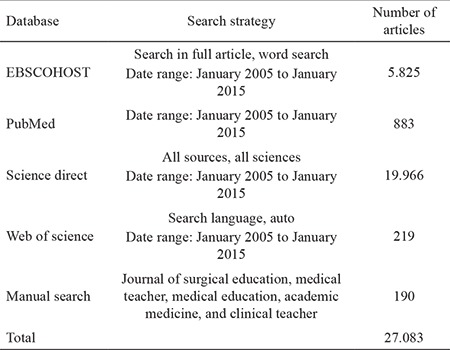
The databases searched and search strategies employed

**Table 3:**
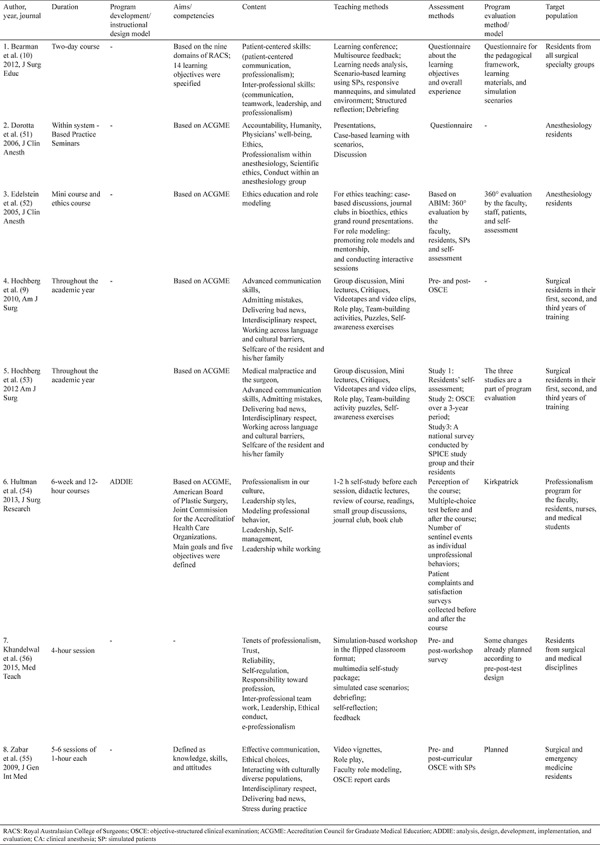
Analysis details of the eight articles on surgical professionalism programs
